# The *Schistosoma mansoni* nuclear receptor FTZ-F1 maintains esophageal gland function via transcriptional regulation of *meg-8*.*3*

**DOI:** 10.1371/journal.ppat.1010140

**Published:** 2021-12-15

**Authors:** Aracely A. Romero, Sarah A. Cobb, Julie N. R. Collins, Steven A. Kliewer, David J. Mangelsdorf, James J. Collins

**Affiliations:** 1 Department of Pharmacology, UT Southwestern Medical Center, Dallas, Texas, United States of America; 2 Department of Molecular Biology, UT Southwestern Medical Center, Dallas, Texas, United States of America; 3 Howard Hughes Medical Institute, Dallas, Texas, United States of America; Texas Biomedical Research Institute, UNITED STATES

## Abstract

Schistosomes infect over 200 million of the world’s poorest people, but unfortunately treatment relies on a single drug. Nuclear hormone receptors are ligand-activated transcription factors that regulate diverse processes in metazoans, yet few have been functionally characterized in schistosomes. During a systematic analysis of nuclear receptor function, we found that an FTZ-F1-like receptor was essential for parasite survival. Using a combination of transcriptional profiling and chromatin immunoprecipitation (ChIP), we discovered that the micro-exon gene *meg-8*.*3* is a transcriptional target of SmFTZ-F1. We found that both *Smftz-f1* and *meg-8*.*3* are required for esophageal gland maintenance as well as integrity of the worm’s head. Together, these studies define a new role for micro-exon gene function in the parasite and suggest that factors associated with the esophageal gland could represent viable therapeutic targets.

## Author summary

Schistosomes kill an estimated 250,000 people every year and cause severe morbidity in millions more. Therefore, it is critical we continue to understand fundamental processes in the parasite so new therapies can be developed. Here, we identify a transcriptional regulator protein SmFTZ-F1 that is critical for parasite survival. We find that this protein controls the levels of a schistosome-specific gene (*meg-8*.*3*) and that *meg-8*.*3* is critical for the maintenance of the worm’s esophageal gland. Loss of \ either meg-8.3 or *Sm*ftz-f1 leads to degeneration of the parasites’ head tissues. These studies suggest that molecules associated with esophageal gland function could represent therapeutic targets.

## Introduction

Schistosomiasis is a disease caused by parasitic flatworms of the genus *Schistosoma* that affects over 200 million people. The main cause of disease pathology is the egg production of the parasites, which can lay several hundred eggs per day while living in the host. The eggs that do not exit into the environment get swept into the circulatory system and lodge in the host’s liver or bladder, depending on the schistosome species, which then leads to inflammation that can disrupt organ function [1].

Currently, praziquantel is the only drug used to treat schistosomiasis and there is a growing need for novel therapeutics [2]. Unfortunately, few processes within these parasites have been shown to be critical for parasite survival, hindering target-based approaches for therapeutic development. Nuclear hormone receptors (NRs) are ligand-activated transcription factors that regulate diverse processes in metazoans [3], including homeostasis, reproduction, and cellular differentiation [4–7]. Given their diverse and conserved roles in metazoan biology, together with a paucity of molecular studies examining their function in schistosomes, we reasoned that examining this class of proteins could reveal biological processes critical to parasite survival.

NRs possess two distinct domains: a zinc finger DNA-binding domain and a ligand-binding domain. These domains, especially the DNA binding domain, are highly conserved across species and can be used to identify genes within the superfamily. In comparing known NR sequences from other metazoan species to schistosome transcriptomic data, we generated a list of 21 putative schistosome NRs. While multiple studies have been conducted on these receptors in *S*. *mansoni* [8–26], their function largely remains uncharacterized in schistosomes. We cloned full-length sequences for 17 out of 21 of these receptors and functionally characterized these receptors using RNA interference and *in situ* hybridizations. We found that mRNA depletion in *S*. *mansoni* of a previously described homolog of NR5A2 (SmFTZ-F1 (Smp_328000)) [12,22] leads to curling of the parasite’s head and eventually death *in vitro*. First identified in *Drosophila*, the FTZ-F1 receptor governs a number of early morphological processes, highlighting its role as a crucial developmental transcription factor [27]. RNA-seq of RNAi-treated parasites revealed transcriptional changes following loss of SmFTZ-F1 and led to the discovery of *meg-8.3* as direct transcriptional target of SmFTZ-F1. *meg-8.3* is expressed exclusively in the worm’s esophageal gland, an enigmatic tissue that has recently been shown to play a critical role in defending the worm from host-attack [28]. We find that loss of either Sm*ftz-f1* or *meg-8.3* results in defects in the maintenance of the esophageal gland and ultimately leads to degeneration of the worm’s head. Together these data suggest that disrupting esophageal gland function is catastrophic for the worm, highlighting the potential of targeting this tissue for the development of therapeutics.

## Materials and methods

### Ethics statement

Experiments with and care of vertebrate animals were performed in accordance with protocols approved by the Institutional Animal Care and Use Committee (IACUC) of UT Southwestern Medical Center (protocol approval number APN 2017–102092).

### Parasite acquisition and culture

Adult S.*mansoni* (6–7 weeks post-infection) were obtained and cultured as previously described [29].

### Molecular biology

cDNAs used for RNAi and *in situ* hybridization analyses were cloned as previously described [30]; oligonucleotide primer sequences are listed (S1 Table). Quantitative PCR analyses to examine knockdown efficiency were performed as previously described [31,32].

### RNA interference

dsRNA was generated as previously described [30,31]. For both the initial NR screen and the RNA-seq “screen” RNAi experiments, RNAi experiments were performed as previously described using paired worms [32]. Subsequent experiments were performed using only male parasites. The parasites were fixed as previously described [31]. Throughout the 20-day experiment, worms were monitored for gross physical phenotypes (*i*.*e*., detachment from the plate, loss of movement, head curling, etc).

### RNAseq for *Smftz-f1* RNAi-treated worms

To examine gene expression changes following loss of Smp_328000 (*Smftz-f1)*, worms were cultured as previously described [33] and only male worms were harvested after 5 or 9 days in culture. We predicted that transcriptional changes that occur at earlier timepoints following RNAi are most likely to represent direct targets of SmFTZ-F1, whereas changes at later time points would represent both the direct and indirect effects of SmFTZ-F1 loss. As controls, worms cultured in parallel were treated with a non-specific dsRNA [31]. For RNA extraction, male worms were collected, excess media removed, and 100 μL of TRIzol was added to the worms. Total cellular RNA was isolated from cells using TRIzol reagent (Invitrogen) following the manufacturer’s protocol and processed for Illumina sequencing. Libraries for RNAseq analysis were mapped to the *S*. *mansoni* genome and analyzed as previously described [33]. RNAseq datasets for the *Smftz-f1*(*RNAi*) experiments are available at NCBI through the accession number (GSE188736).

To define potential SmFTZ-F1 binding sites, we extracted genome sequences 2kb upstream of genes down-regulated at both D5 and D9 following *Smftz-f1*(*RNAi*) using bedtools (https://bedtools.readthedocs.io/en/latest/index.html) and searched for a variant of the NR5A2 motif (http://jaspar.genereg.net/matrix/MA0505.1) using FIMO (version 5.0.1)[34,35].

### Purification of Recombinant SmFTZ-F1

To generate an anti-SmFTZ-F1 antibody, an N-terminal fragment of SmFTZ-F1 corresponding to AA 1–150 was amplified and subcloned into the pET28 vector with an N-terminal SUMO-His6 tag for expression in *Escherichia coli*. This fragment was purified from transformed BL21 cells grown in LB medium and induced with 1mM Isopropyl-β-D-thiogalactoside for 16 hrs at 18°C. Cells were pelleted and resuspended into lysis buffer containing 50 mM NaH_2_PO_4_, 500 mM NaCl, 20 mM Imidazole, and protease inhibitors (Roche cOmplete, Mini, EDTA-free). The suspension was sonicated, lysate was centrifuged for 25 min at 14,000 x g, and rotated with resin for 1 hr. The resin was washed, and protein was eluted in lysis buffer containing 250mM imidazole. The sample was then dialyzed to remove the NaCl and imidazole. The SUMO-His6tag was then cleaved using purified ULP-1. The protein was then concentrated using an Amicon concentrator, 10-kDa cut-off (Millipore). Rabbit polyclonal antibodies were generated by YenZym (California).

### Imaging

Confocal imaging of fluorescently labeled samples and brightfield imaging (*i*.*e*, whole mount in situ hybridizations) were performed using a Nikon A1 Laser Scanning Confocal Microscope or a Zeiss Axio Zoom V16 equipped with a transmitted light base and a Zeiss Axio Cam 105 Color camera, respectively.

### Electrophoretic mobility shift assay (EMSA)

Biotin end-labeled probes were prepared using Pierce Biotin 3’ End DNA Labeling Kit following manufacturer’s instructions with the following modifications. Complementary oligos were end-labeled separately and then annealed before use. Oligos were annealed by mixing together equal amounts of labeled complementary oligos, denaturing the mixture at 90°C for 1 minute, then slowly cooling and incubating at the melting temperature for 30 minutes. The oligos were then frozen and thawed on ice for use. SmFTZ-F1 protein was prepared by *in vitro* translation with PURExpress (NEB). EMSA was performed using the LightShift Chemiluminescent EMSA Kit (ThermoFisher Scientific, USA) according to the manufacturer’s instructions. DNA binding reactions were performed in a 20  μL volume containing biotin-labeled oligonucleotides and *in vitro* translated protein from the PURExpress kit. Reaction products were then separated by electrophoresis. Thereafter, the protein-DNA complexes were transferred onto a positively charged nylon membrane (Millipore, USA) and detected by chemiluminescence using a GE Healthcare ImageQuant LAS 4000. For competitive binding experiments, 20- or 200-fold excesses of unlabeled DNA probes were also included in the binding reaction.

### Western blotting to detect SmFTZ-F1

To generate protein lysates, RNAi-treated male parasites were separated with 0.5% tricaine, homogenized in 100 μl of sample buffer (2.5 mL of Solution D (0.47 M Tris, pH 6.7 (with 1 M phosphoric acid)) 1 mL of 100% glycerol, 1 mL 20% SDS, 0.5 mL H_2_O, and 50 μL of 1 M DTT) and protease inhibitors (Roche cOmplete, Mini, EDTA-free)). Homogenized samples were sonicated using a Biorupter system at 30% amplitude for 5 minutes, 0.5 sec on, 0.5 sec off. Protein concentrations were determined by Pierce Detergent Compatible Bradford Assay Kit. 40 mg of lysate was separated by SDS-PAGE, proteins were transferred to nitrocellulose membranes, membranes were blocked in Li-Cor Odyssey Blocking Buffer, incubated in rabbit anti-SmFTZ-F1 (0.5 mg/mL) and mouse anti-Actin (0.05mg/ml, Monoclonal JLA20, Developmental Studies Hybridoma Bank), washed in TBST, and incubated in secondary antibodies (1:10,000 goat anti-rabbit IR Dye 680 RD, 1:15,000 goat anti-mouse IgM IR Dye 800CW, Li-Cor). Blots were imaged using a Li-Cor Odyssey Infrared Imager.

### Immunofluorescence

The parasites were fixed in methacarn (60 mL of methanol, 30 mL chloroform, and 10 mL glacial acetic acid) for 1 hr. Parasites were dehydrated in MeOH and stored at −20°C until processing. Worms were then processed similarly to colorimetric and fluorescence *in situ* hybridization following rehydration steps which were performed as previously described [23,29,31] with the following modifications. After post-fixation with 4% formaldehyde in PBSTx, the worms were then washed twice in PBSTx then were blocked with FISH block (0.1 M Tris pH 7.5, 0.15 M NaCl and 0.1% Tween-20 with 5% Horse Serum and 0.5% Roche Western Blocking Reagent [36]) for 1 hr and incubated overnight with 2.5 μg/mL of anti-SmFTZ-F1 antibody. After 6X PBSTx washes for 20 minutes each, the worms were then incubated in secondary antibody (1:1000 Goat anti-Rabbit IgG (H+L) Secondary Antibody, Alexa Fluor 488 conjugate) in FISH block for 4 hours at 4°C. After several washes in PBSTx, parasites were counterstained with DAPI for 30 minutes then placed in 80% glycerol and were then mounted on slides in Vectashield (Vector Laboratories).

### ChIP-qPCR

ChIP assays were performed using SimpleChIP Plus Enzymatic Chromatin IP Kit (Cell Signaling) according to the manufacturer’s instructions. Before harvesting, male worm heads were amputated and processed for chromatin immunoprecipitation. Briefly, worms were fixed with formaldehyde (0.75%) to cross-link DNA and proteins, DNA was digested using micrococcal nuclease, chromatin was sheared using a Biorupter (8 cycles of sonication: 30s each, 0.5 sec on, 0.5 sec off; amplitude = 30%,), chromatin was incubated with antibodies (SmFTZ-F1 at 5 μl, preimmune serum negative control at 5 μl), and immunoprecipitates were bound to protein G magnetic beads (30 μl). The protein-DNA cross-linking was reversed, DNA was purified, and enrichment of DNA sequences was detected using qPCR and primers listed in S1 Table. Data were normalized and analyzed using percent input analysis [37].

### FISH/WISH using RNAi treated worms

Colorimetric and fluorescence *in situ* hybridization analyses were performed as previously described [23,29,31]. Dextran labeling of the parasite gut was done as previously described [23]. All fluorescently labeled parasites were counterstained with DAPI (1 μg/ml), cleared in 80% glycerol, and mounted on slides with Vectashield (Vector Laboratories).

### Carmine staining

Carmine red staining was performed as previously described [38]. Confocal laser scanning microscopy images were taken on a Nikon A1 Laser Scanning Confocal Microscope.

### Statistical analysis

All two-way comparisons were analyzed using Student’s t-test. RNAseq data was analyzed using DESeq2 [39].

## Results/discussion

### An SmFTZ-F1 homolog is essential for parasite vitality

To investigate potential roles that nuclear receptors play in schistosome biology, we set out to examine their expression and function using both whole mount *in situ* hybridization (WISH) and RNA interference (RNAi). We cloned either full or partial length cDNAs for 17 of 21 receptors from adult *S*. *mansoni* cDNA. We were unable to clone the remaining four due to low expression in the adult stage [8]. Expression analyses revealed that a majority of the NRs were expressed in the reproductive systems of both the males and females (S1 Fig). VF1, a parasite-specific NR [40], was expressed in cells of the female vitellaria (a tissue responsible for making “yolk” cells) as well as what appeared to be primordial vitellaria of male worms (S1 Fig). Although many NRs were broadly expressed throughout the parasite tissues (*e*.*g*., *Smftz-f1*), the expression of some NRs was restricted to smaller sets of cells (e.g., SmNR4A4 and SmCOUP-TF1).

**Fig 1:**
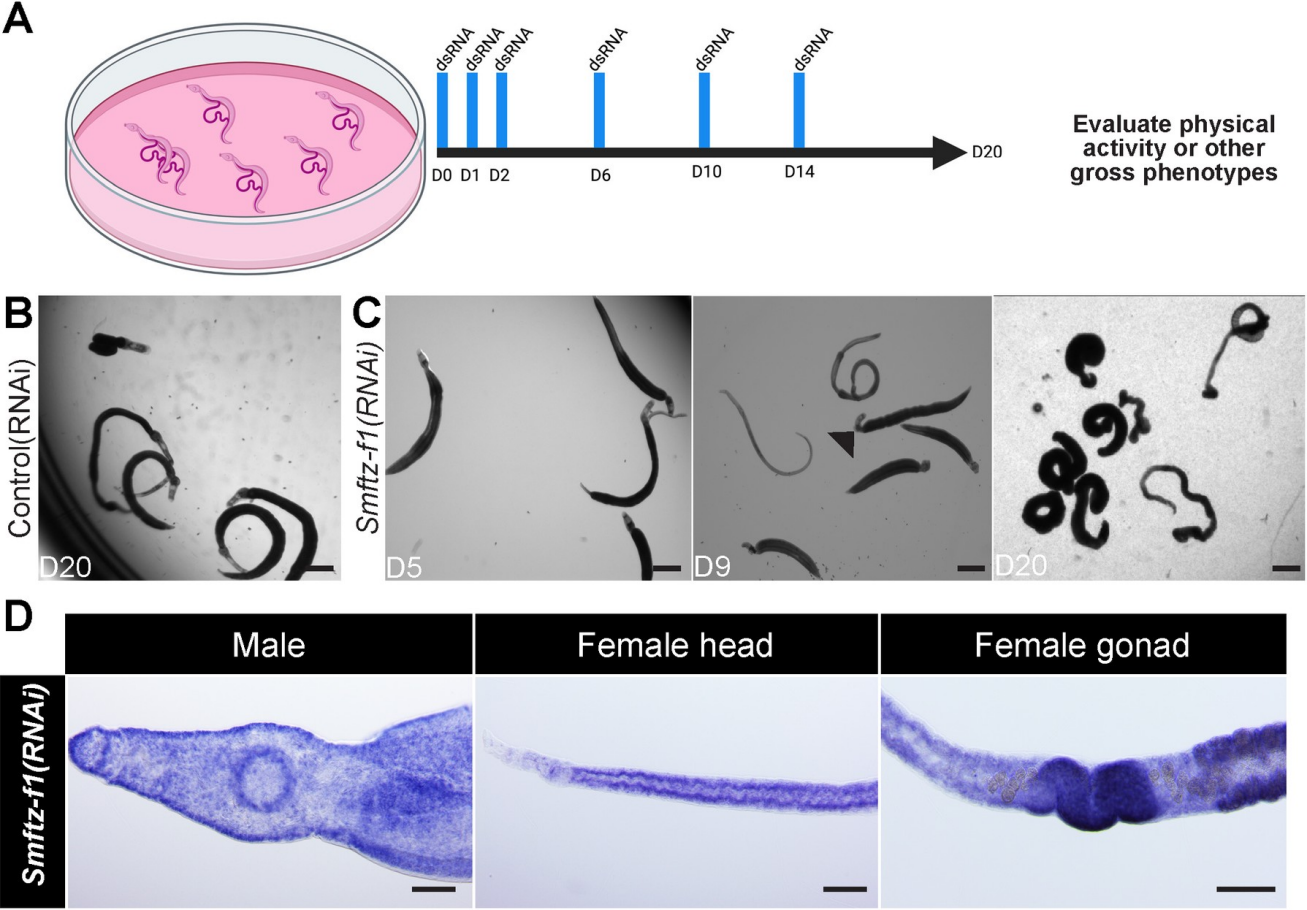
An SmFTZ-F1 homolog is essential for parasite vitality. **(A)** A schematic diagram depicting RNAi screen strategy **B and C**, Images of control and *Smftz-f1(RNAi)* worms during *in vitro* culture. At day 20 of *in vitro* culture, control(RNAi) animals remain as male and female worm pairs, are firmly attached to the bottom of the plate, and move freely. By D9 of *in vitro* culture, *Smftz-f1(RNAi)* animals become unpaired and fail to firmly attach to the bottom of the place. Over time, movement of *Smftz-f1(RNAi)* worms diminishes and they stop moving altogether. At D9, the heads are curled (arrow). At D20 the worm’s full body is curled. *n* = 10 worm pairs, *>* 3 biological replicates **(D)** Whole mount *in situ* hybridization showing mRNA expression of *Smftz-f1* in both male and female parasites. The expression is found throughout the bodies of both sexes and does not seem to be specific for a tissue. Scale Bars: B and C, 500 μm. D,100 μm.

To explore the function of the schistosome NR complement, we performed RNAi for each NR and examined whether any of these treatments compromised parasite vitality *in vitro* (Fig 1A). We found that RNAi-mediated depletion of the mRNA encoding the schistosome *ftz-f1* homolog [12,22] resulted in a fully penetrant degenerative phenotype (Fig 1B and 1C). By day 9 (D9), male and female *Smftz-f1(RNAi*) parasites became unpaired and lost the ability to attach to the surface of the tissue culture dish, and by D20, movement in *Smftz-f1(RNAi)* parasites was virtually nonexistent in male worms (S1 Video). The mRNA of *Smftz-f1* was found to be expressed throughout both sexes of the worm (Figs 1D and S2A). Similar phenotypes were observed with dsRNAs targeting two distinct regions of the *Smftz-f1* gene, indicating these effects are specific to the reduction of *Smftz-f1* level and not due to off-target effects (S2B–S2D Fig).

### RNAseq reveals possible direct targets of SmFTZ-F1

**Fig 2:**
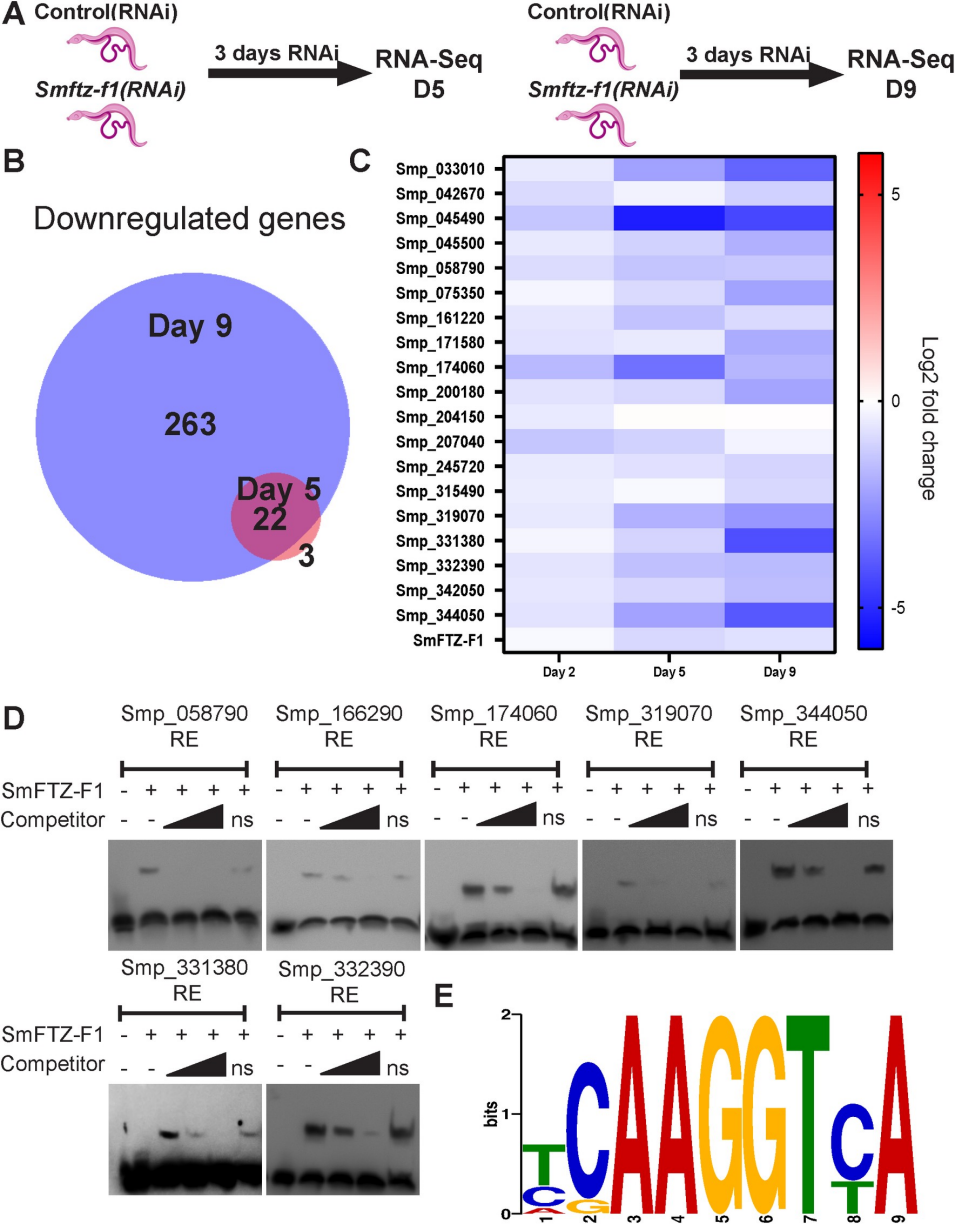
RNAseq reveals possible direct targets of SmFTZ-F1. (**A)** Strategy for RNAseq, paired worms were subsequently separated and only male parasites were used. **(B)** Venn diagram of down-regulated RNAseq gene sets from Day 5 and 9. **(C)** Heat map of the 20 most differentially down-regulated genes following *Smftz-f1*(RNAi) treatment. *n = 10 worms*, 3 biological replicates. **(D)** Micrographs of EMSAs using biotin-labeled oligonucleotides corresponding to each of the candidate SmFTZ-F1 response elements. EMSAs were performed in presence or absence of *in vitro* translated SmFTZ-F1 protein ± the presence of 20-fold or 200-fold direct unlabeled competitor oligonucleotides. ns, non-specific oligo at 200-fold excess. *n =* 2 biological replicates. **(E)** Predicted DNA-binding motif sequence for nuclear receptor SmFTZ-F1. The height of the letter represents how frequently that nucleotide is observed in that position. The logo was generated using the MEME Suite.

NRs are DNA-binding transcriptional regulators [41]. Therefore, we reasoned we could use transcriptional profiling to identify potential direct transcriptional targets and gain a deeper insight into the molecular changes responsible for the *Smftz-f1*(*RNAi*) phenotype. For these studies, we compared the transcriptional profile of control and *Smftz-f1(RNAi)* parasites at timepoints before (“early” D5 post-RNAi) and after (“late” D9 post-RNAi) we observed the degenerative changes in the *Smftz-f1(RNAi)* treatment group. We predicted that transcriptional changes that occur at the “early" timepoint following RNAi are most likely to represent direct targets of SmFTZ-F1, whereas changes at the “late” time point would represent both the direct and indirect effects of SmFTZ-F1 loss (Fig 2A). Since FTZ-F1 orthologs in other systems are transcriptional activators [34,42,43], we focused on genes down-regulated following RNAi. As predicted, on D5 following *Smftz-f1(RNAi*) treatment we noted that a relatively small number of genes (25) were down-regulated; this number increased to 263 genes by D9 of RNAi treatment (Fig 2B) (log_2_ fold-change -1.5, *p* < 0.01)(S2 Table). Importantly, 22 of the 25 genes down-regulated on D5 were also down on D9 (Fig 2B). qPCR validation studies of the 20 most downregulated of these 25 genes confirmed our observations from RNAseq for 19 out of 20 genes (Fig 2C, S3 Table).

**Fig 3:**
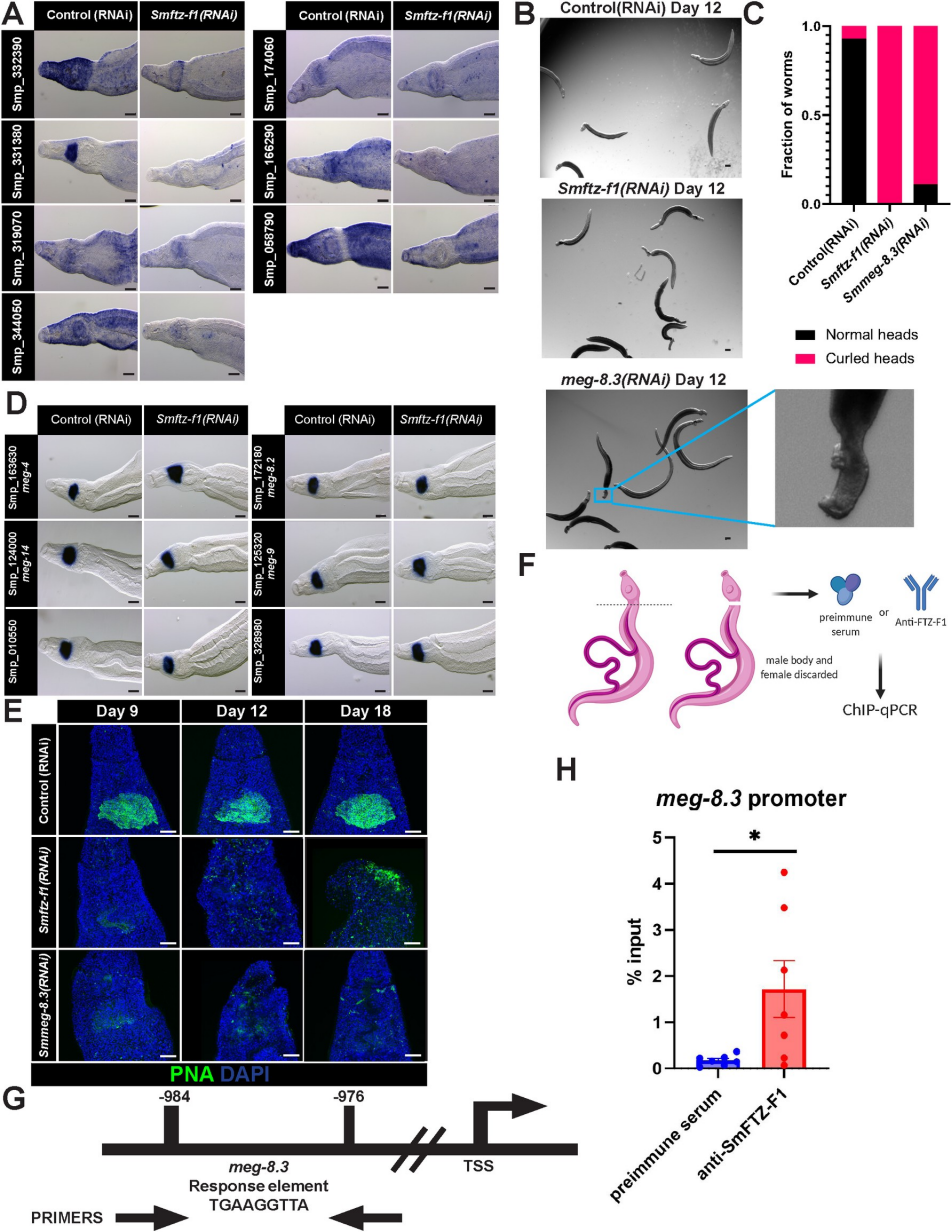
*meg-8*.*3* is a putative target of SmFTZ-F1. **(A)** Whole mount *in situ* hybridizations for putative SmFTZ-F1 target genes on D9 of RNAi. Smp_331380 is exclusively expressed in the worm esophageal gland. *n =* 10 worms, 3 biological replicates. **(B)** Images of control, *Smftz-f1* and *meg-8*.*3* RNAi-treated worms during *in vitro* culture. Control(RNAi) animals remain firmly attached to the bottom of the plate, and move freely. At D12 of *meg-8*.*3(RNAi)* animals fail to attach to the bottom of the culture plate. Their heads begin to curl inward on themselves (see inset). *n >* 10 worms, 3 biological replicates. **(C)** Plots depicting the number of animals that display normal or curled phenotypes following RNAi treatment at D15. **(D)** Whole mount *in situ* hybridization of D9 *Smftz-f1* RNAi-treated worms using known esophageal gland markers. Control and RNAi-treated worms show no difference in esophageal gland staining. *n >* 10 worms, 3 biological replicates. (**E**) PNA labeling (green) of esophageal gland using RNAi treated worms at days 9, 12, 18. Maximum intensity projections are shown. *n >* 10 worms, 3 biological replicates (**F**) Cartoon of the ChIP-qPCR experiment **(G)** Schematic of the 2kb upstream region of the *meg-8*.*3* promoter with the sequences and position of the response element and primer set used, length not proportional to the size. **(H)** ChIP-qPCR, shown as percentage of input DNA, for SmFTZ-F1 or preimmune serum antibodies at the putative promoter of *meg-8*.3. Data are mean ± SEM from 7 biological replicates. *p<0.05 by Student’s t-test. Scale bars, A,B,D, 100μm. E, 50 μm.

Previous studies using Electrophoretic Mobility Shift Assays (EMSA) showed that SmFTZ-F1 can bind to a human SF-1 response element [12]. To examine whether this sequence could be found in the regulatory regions of genes down-regulated at both D5 and D9, we searched 2kb upstream of the transcription start site of each gene for a related response element using FIMO [34,35]. This analysis revealed 64 putative SmFTZ-F1 response elements present upstream of 22 genes. We examined 30 of these putative response elements by EMSAs to determine which of these elements were bound by SmFTZ-F1 (S4 Table, S3 Fig). These studies revealed that SmFTZ-F1 bound efficiently to sequences upstream of seven genes (Fig 2D), suggesting a potential consensus binding sequence for SmFTZ-F1 (Fig 2E). We examined these seven genes in more detail by performing WISH on control and *Smftz-f1*(*RNAi*)-treated parasites. Consistent with our RNAseq data (Fig 2B) and qPCR studies (Fig 2C), each of the seven genes was markedly downregulated following RNAi treatment (Fig 3A). With the exception of Smp_331380, which was expressed specifically in the esophageal gland, these genes were broadly expressed throughout the worm, and their expression appeared to be uniformly reduced by *Smftz-f1* knockdown. This observation is consistent with SmFTZ-F1 regulating gene expression across several cell types.

### *Smftz-f1* and *meg-8*.*3* share similar RNAi phenotypes

We could envision two non-mutually exclusive scenarios for the phenotype observed following *Smftz-f1* knockdown. In the first scenario, loss of *Smftz-f1* disrupts the expression of multiple target genes and this broad transcriptional misregulation results in parasite degeneration and death. Alternatively, loss of *Smftz-f1* leads to misregulation of individual target genes resulting in some or all of the observed phenotype. To distinguish between these models, we performed RNAi on each of the seven of the putative *Smftz-f1* target genes. Consistent with the second scenario, we observed that RNAi of Smp_331380 led to detachment of parasites from the tissue culture dish beginning at D12 and a head curling phenotype identical to that observed following *Smftz-f1*(*RNAi*) (Fig 3B and 3C).

Based on BLAST homology, Smp_331380 appeared to be a member of the schistosome-specific MEG-8 protein family, sharing complete sequence identity with the previously described MEG-8.3 protein [44,45]. Thus, we will refer to Smp_331380 as *meg-8*.*3* from this point forward. MEG proteins are small proteins of largely unknown function that are encoded by small exons (~ 6 to 36 bp) that were uncovered during the sequencing of the *S*. *mansoni* genome [46,47]. The *meg-8* protein family includes four paralogs (*meg-8*.*1*, *8*.*2*, *8*.*3*, and *8*.*4*), whose homolog in *Schistosoma japonicum* MEG-8.2 appears to interact with host leukocytes as they pass by the worm’s esophageal gland [48]. The *meg-8*.*3* gene is predicted to encode an 11 kD protein with a predicted signal peptide (S4A Fig) generated from 10 exons [49], some of which are as small as 15 bp in length (S4B Fig).

In principle, the observed loss of *meg-8*.*3* expression in the esophageal gland following *Smftz-f1* loss could be due to either misregulation of *meg-8*.*3* expression or loss of the esophageal gland tissue entirely. To explore this, we queried scRNAseq data [23] and examined the expression of a panel of genes expressed in the esophageal gland following *Smftz-f1*(*RNAi*) treatment. In contrast to *meg-8*.*3* expression that was rapidly depleted following *Smftz-f1* knockdown (Fig 3A), the expression of six esophageal gland marker genes were not obviously different in *Smftz-f1(RNAi)* parasites (Fig 3D). This suggests that loss of *Smftz-f1* appears to rapidly and specifically lead to the reduction of *meg-8.3* expression in the esophageal gland.

We next evaluated the general morphology of the esophageal gland using the lectin PNA [50]. Under control conditions, the worms maintained a normal esophageal gland, however loss of either *Smftz-f1* or *meg-8*.*3* resulted in a loss of PNA staining in the esophageal gland with little labeling observed by D18 of RNAi (Fig 3E). Simultaneously, we noted a significant reduction in the expression of the esophageal gland marker, *meg-4* between *D15 and D18 of meg-8*.*3* RNAi treatment (S4C and S4D Fig). Taken together, these data are consistent with the model that SmFTZ-F1 regulates the expression of *meg-8*.*3* in the esophageal gland, and that loss of *meg-8*.*3* expression in the tissue results in loss of esophageal gland function that is at least partially responsible for the detachment and head curling observed in *Smftz-f1(RNAi)* parasites.

Given that the *S*. *mansoni* genome contains three other MEG-8 proteins with sequence identity to MEG-8.3, we wanted to rule out the possibility that our phenotype was due to off-target effects. Therefore, we measured the levels of *meg-8*.*3* and its closest relative *meg-8*.*2* following *meg-8*.*3* RNAi. As anticipated, RNAi of *meg-8*.*3* resulted in large decreases in *meg-8*.*3* mRNA levels (S4E Fig). Quantitative analysis of genes expressed in the esophageal gland following *meg-8*.*3*(RNAi) is complicated by the fact that loss of *meg-8*.*3* appears to lead to loss of esophageal gland tissue over time (Figs 3E, S4C and S4D). Therefore, to examine the potential of RNAi off-target effects on *meg-8*.*2* levels following *meg-8*.*3* RNAi, we examined the expression of *meg-8*.*2* and an unrelated esophageal gland marker gene *cystatin* (Smp_328980) [23]. When compared to *meg-8*.*3* levels, we observed modest decreases in the levels of both *meg-8*.*2* and *cystatin* following *meg-8*.*3*(RNAi) (S4E Fig), suggesting the reduction in transcript levels is due to tissue dysfunction rather than off target RNAi effects. This observation, along with the fact *meg-8*.*2* and *meg-8*.*3* share limited sequence identity on the nucleotide level [51], indicates that *meg-8*.*3* RNAi-treatment specifically targets *meg-8*.*3* levels.

### SmFTZ-F1 controls *meg-8*.*3* expression

To determine if *meg-8*.*3* is indeed a direct target of SmFTZ-F1 in the parasite, we generated an antibody against SmFTZ-F1 and performed chromatin immune precipitation experiments. Our SmFTZ-F1 antiserum recognizes an ~110 kD protein in whole worm lysates (S5A Fig) and the levels of this protein are depleted upon *Smftz-f1* dsRNA treatment (S5B Fig), confirming the specificity of the antibody. As expected for a DNA-binding transcription factor, we observed by immunofluorescence that this antibody labeled nuclei throughout the worm body (S5C Fig). This signal was also lost following *Smftz-f1*(*RNAi*) treatment (S5D Fig).

Given the specificity of the anti-SmFTZ-F1 antibody, we set out to determine if *meg-8.3* is a direct target of SmFTZ-F1 in vivo by ChIP. For these studies, we amputated the heads of male worms to enrich for cells expressing *meg-8.3*, performed ChIP using either anti-SmFTZ-F1 or pre-immune serum, and then used quantitative PCR to evaluate relative SmFTZ-F1 binding to various sites in the genome (Fig 3F and 3G). As an initial step, we performed various controls evaluating SmFTZ-F1 binding at distal regions 5 and 10 kb upstream of *meg-8.3* and at a region surrounding a SmFTZ-F1 consensus response element found upstream of *foxA*, a gene encoding a transcription factor that appears to be a master regulator of esophageal gland identity [28]; we did not note significant SmFTZ-F1 binding to any of these sites (S6A–S6D Fig). We next evaluated the consensus SmFTZ-F1 response element upstream of *meg-8.3* that was bound by SmFTZ-F1 *in vitro* (Fig 2D). Evaluation of the sequence surrounding this consensus site revealed that it was repeated at several sites within the *S. mansoni* genome (S1 File), ruling out possibility of designing primers that could specifically amplify this region of the genome. However, since a large number of transcription factor binding sites are known to exist in repetitive elements *[52–54]*, we proceeded with ChIP studies. Using qPCR primers that amplify the region surrounding the putative SmFTZ-F1 binding site upstream of *meg-8.3*, we noted a statistically-significant 10-fold increase when we performed ChIP using the anti-SmFTZ-F1 (Fig 3H). ChIP-qPCR using *Smftz-f1* RNAi-treated worms showed that the enrichment of SmFTZ-F1 at the response element in the *meg-8.3* promoter was abolished to levels similar to those observed using preimmune serum (S6E Fig). These data, along with our RNAseq studies (Fig 2B) and EMSA data (Fig 2D), strongly support the model that SmFTZ-F1 binds to a response element found in the *meg-8.3* promoter.

### MEG-8.3 is essential for the maintenance of head tissue

**Fig 4:**
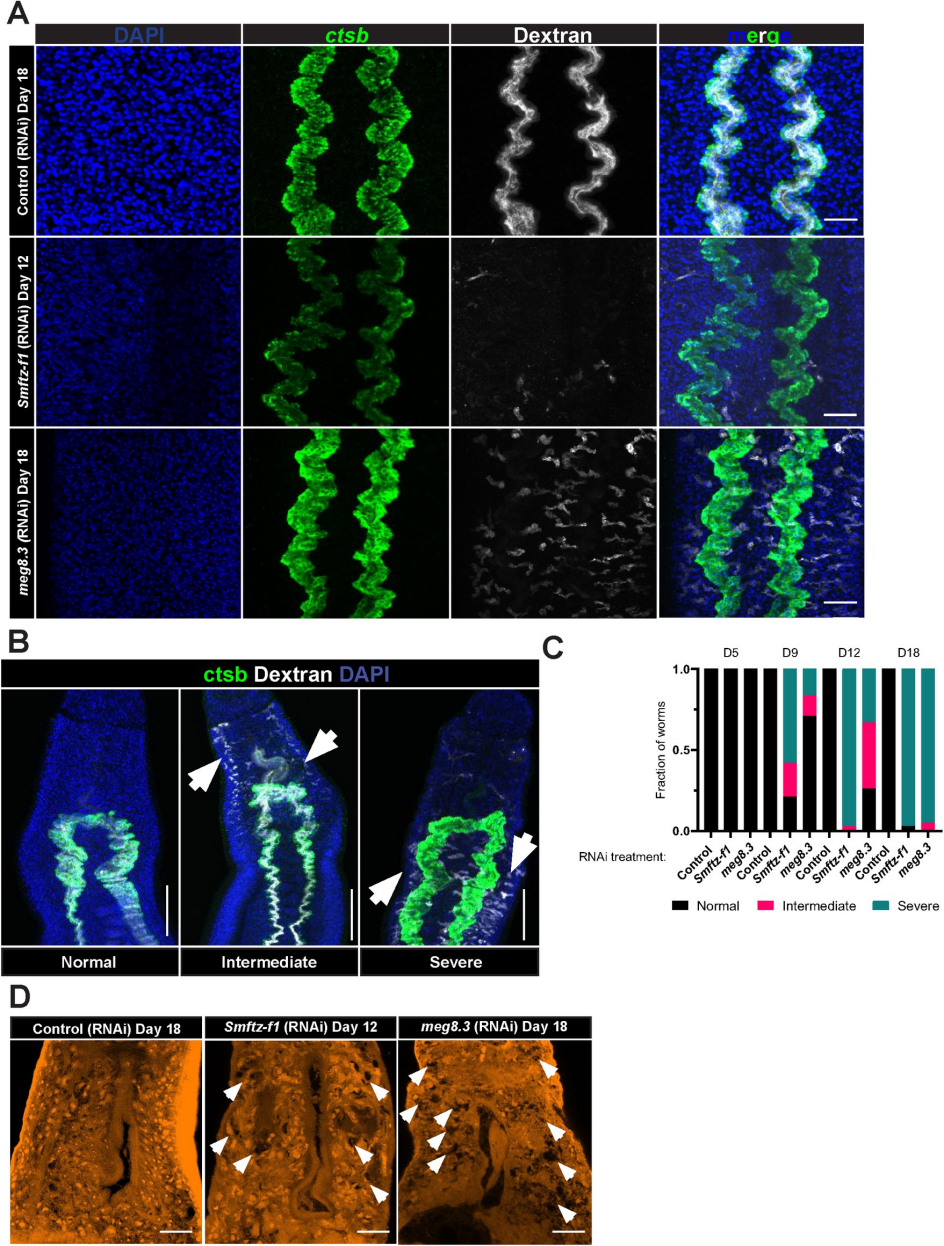
*meg-8*.*3* is essential for esophageal gland tissue maintenance. **(A)** FISH of the gut marker *ctsb* (green), and fluorescent dextran (white) in the heads of control(RNAi), *Smftz-f1(RNAi)* and *meg-8*.*3(RNAi)* animals. Representative of >10 animals from 3 experiments **(B)** Three distinct phenotypic severities are observed following RNAi: Normal, Intermediate, and Severe. Normal animals possess an intact and well-organized gut as observed by *ctsb* expression and fluorescent dextran in lumen of the gut. Intermediate animals have normal gut structure and possess fluorescent dextran in the gut, but also contain dextran collecting in the protonephridial system in the head (arrows). Severe animals possess a relatively normal gut structure and very little, if any, dextran labeling in the gut. Instead, dextran labeling is found in the protonephridial system (see arrows). **(C)** Plots depicting the relative fraction of animals that display Normal, Intermediate, or Severe phenotypes at D5–D18 of RNAi. >12 animals from three separate experiments were observed for each time point. **(D)** Carmine-stained head regions of control(RNAi), *Smftz-f1(RNAi)*, and *meg-8*.*3(RNAi)* treated worms at different time points (Arrows highlight “holes”). Representative of >10 animals from 4 experiments. Scale Bars: A, D 25 μm; B,100 μm.

Our data show that SmFTZ-F1 maintains the expression of *meg-8*.*3*, and loss of *meg-8*.*3* expression results in profound morphological changes in the male head. Given the specific expression of *meg-8*.*3* in the esophageal gland, we predicted that this phenotype could be attributed to defects in esophageal gland function. The esophageal gland appears to be critical for schistosome feeding and the passage of materials (*e*.*g*., cells) into the intestine [55]. To explore this, we cultured *Smftz-f1* and *meg-8*.*3*(*RNAi*) parasites in fluorescent dextran, which the worms ingest and eventually absorb into their intestine [23,56], and performed FISH for *cathepsin B* (*ctsb)* to assess gut structure [23]. Following 12 hours of culture with dextran, we noted that control parasites assimilated the dextran into their intestine (Fig 4A). In contrast, we noted varying rates of dextran uptake in parasites between 5 to 18 days of *Smftz-f1* and *meg-8*.*3(RNAi*) treatment. At early time points, *Smftz-f1* and *meg-8*.*3(RNAi*) parasites were indistinguishable from controls. However, as the experiment continued, *Smftz-f1* and *meg-8*.*3* knockdown parasites progressively lost the ability to accumulate dextran in their intestine (Fig 4B and 4C).

We also noted that as *Smftz-f1* and *meg-8*.*3* knockdown parasites failed to accumulate dextran in their intestine, they began accumulating the label in their protonephridial (excretory) system (Figs 4A and S7). The protonephrida are a network of ciliated and non-ciliated tubules that collect materials from the worm’s tissues and excrete them via a pore at the posterior of the worm [55]. Thus, we reasoned that the dextran may permeate the tissues of *Smftz-f1* and *meg-8*.*3* knockdown worms and subsequently be collected in the protonephridal system. One potential mechanism to explain the presence of dextran in the worms inner tissues is that loss of SmFTZ-F1*/*MEG-8.3 results in degenerative changes that result in an inability of the worms to maintain barrier function. To examine this, we used carmine red straining to examine tissue integrity focusing on the worm’s head since this is where the *meg-8*.*3* RNAi phenotype was localized. Consistent with *Smftz-f1* and *meg-8*.*3* being critical for tissue integrity in the worm head, we noted significant differences in head morphology between control and *Smftz-f1* and *meg-8*.*3* RNAi parasites. While controls maintained their head tissues and cellular architecture, *Smftz-f1* (on D12) and *meg-8*.*3* (on D18) RNAi treatment resulted in notable disruption in tissue morphology that manifested as “holes” in the heads of the parasites.

## Conclusions

Microexon genes have been cataloged across schistosome species and their expression appears to be mainly (but not exclusively) associated with the esophageal gland [44,49,57]. Beyond the fact that most microexon genes are predicted to encode secreted or membrane-anchored proteins in the worms [44,49,57], their molecular functions remain largely elusive. Our data suggest that disruption of either *Smftz-f1* or its transcriptional target *meg-8*.*3* (Fig 3) results in tissue degeneration, which in turn prevents normal worm attachment (Fig 3B and 3C), feeding (Fig 4A, 4B and 4C), and barrier function (Fig 4D). Thus, control of *meg-8*.*3* expression in the esophageal gland appear critical for the parasite.

Recent studies have highlighted the importance of the esophageal gland not only in blood feeding but also as a central player in the parasite’s ability to evade host immunity [28,58]. Indeed, loss of the transcription factor *foxA* results in esophageal gland loss, blunting the ability of parasites to survive in immunocompetent, but not immunocompromised, hosts [28]. Curiously, *foxA(RNAi)* parasites appear phenotypically normal during extended *in vitro* culture. This is in contrast to what we observe when *meg-8*.*3* is depleted from the esophageal gland by RNAi (Fig 3B). Since loss of *foxA* results in loss of all examined esophageal gland markers, and presumably *meg-8*.*3* as well, it would appear that loss of *meg-8*.*3* can be tolerated as long as it occurs in the context of the depletion of other esophageal gland-specific factors. We speculate that MEG-8.3 acts in an inhibitory fashion to blunt the activity of other esophageal gland-expressed factors, and when MEG-8.3’s inhibitory activity is lost, these factors have destructive effects on the parasite’s tissues. Thus, when the expression of *meg-8*.*3* is lost simultaneously with other esophageal gland-specific factors (e.g., following *foxA* RNAi) these destructive effects are mitigated. Exploring the biochemical function of *meg-8*.*3* and defining potential interacting partners will be essential for addressing this issue in the future.

Taken together, our data not only highlight the critical role for SmFTZ-F1 in controlling *meg-8.3* expression but also demonstrate that perturbing the function of a single esophageal gland-specific factor can have deleterious effects on the parasite. Because many esophageal gland-specific factors appear to be parasite specific [44,47], targeting factors associated with this tissue could yield highly-selective therapeutics. Therefore, establishing a deeper understanding the esophageal gland could result in new classes of drugs to treat schistosomiasis.

## Supporting information

S1 Fig Nuclear receptors are expressed differentially between the male and female parasite. Whole mount *in situ* hybridization showing mRNA expression of NRs in male and female parasites. Anterior faces left. Representative of >10 animals from 3 biological replicates. Scale Bars: 100μm.
(TIFF)

S2 Fig SmFTZ-F1 is expressed in all cell types and can be knocked down using RNAi. **(A)** UMAP plot of gene expression of SmFTZ-F1 (Smp_328000), plot generated using Schistocyte [23] (**B)** Graph of relative quantification of SmFTZ-F1 as determined by qPCR in either control(RNAi) or *Smftz-f1(RNAi)* animals. Data are mean ± SD from 3 biological replicates. *p<0.05 by Student’s t-test. (**C)** Cartoon of *Smftz-f1* cDNA (top) and cDNA regions (in AA) targeted by two independent RNAi constructs (pAR22 and pAR31). pAR22 contains a cDNA fragment that spans from AA 360–700 of the *smftz-f1* cDNA. pAR31 contains a cDNA fragment that spans from AA 1–352 of the *smftz-f1* cDNA **(D)** Images of alternate *Smftz-f1(RNAi)* worms during *in vitro* culture. By D9 of *in vitro* culture, *Smftz-f1(RNAi)* animals fail to firmly attach to the bottom of the place. At D14, the worms are beginning to curl. Scale bar, 1 mm.
(TIFF)

S3 Fig Electrophoretic mobility shift assays (EMSAs) with biotin-labelled oligonucleotides corresponding to each of the candidate SmFTZ-F1 response elements. EMSAs were performed in presence or absence of *in vitro* translated SmFTZ-F1 protein ± the presence of 20-fold and 200-fold direct unlabeled competitor or 200-fold excess unlabeled competitor oligonucleotides.
(TIFF)

S4 Fig *meg-8*.*3* is part of the MEG family of proteins and is found in the esophageal gland. **(A)** Amino acid sequence of MEG-8.3. Red indicates a signal peptide according to SignalP **(B)** Schematic representation of gene structure of *meg-8*.*3*. Exon boxes are proportional to their lengths in bp. Lines represent introns shown with a length not proportional to their size. (**C**) Double FISH for *meg-8*.*3* and *meg-4* at day 15 for control(RNAi), *Smftz-f1(RNAi)*, and *meg-8*.*3(RNAi)* parasites. Maximum intensity projections shown. *n =* 3 biological replicates of >5 worms per experiment. (**D**) Double FISH for *meg-8*.*3* and *meg-4* at day 18 for control(RNAi) and *meg-8*.*3(RNAi)* parasites. Maximum intensity projections shown. *n =* 1 replicate, 5 worms per treatment. **(E)** Graph of relative quantification of *Smmeg-8*.*3*, *Smmeg-8*.*2*, Smp_328980 (cystatin) as determined by qPCR in either control(RNAi) or *Smmeg-8*.*3(RNAi)* animals at Day 7 after the first dsRNA treatment. Data from 3 biological replicates. *p<0.05, **p<0.01, ***p<0.0001 by Student’s t-test. Error bars represent 95% confidence intervals. Scale bars: D, 50 μm. E, 25μm.
(TIFF)

S5 Fig Validation of antibody to SmFTZ-F1. **(A)** Immunoblot of worm lysate testing the specificity of the SmFTZ-F1 antibody. Full length SmFTZ-F1 is predicted to be ~110 kD. **(B)** Western blot showing depletion of SmFTZ-F1 protein levels following *Smftz-f1*(*RNAi*). Actin labeling a positive control. *n =* 7 biological replicates taken from ~10 male worms/replicate **(C)** Immunofluorescence demonstrating that SmFTZ-F1 protein is found in the nuclei of cells. Maximum intensity projection (60X) is shown. *n =* 3 biological replicates of at least 5 worms per replicate. **(D)** Immunofluorescence of Day 12 control(RNAi) or *Smftz-f1(RNAi)* worms showing depletion of SmFTZ-F1^+^ cells *n =* 10 worms, 3 biological replicates. Scale bars: D, 5μm. E, 25μm.
(TIFF)

S6 Fig Several genomic regions do not display SmFTZ-F1 binding. **(A)** Schematic of the 10kb upstream region of the Smp_331380 promoter with the position of the primer sets used. Length not proportional to the size. (**B)** ChIP-qPCR, shown as percentage of input DNA, for SmFTZ-F1 or preimmune serum antibodies at control regions 10kb or 5kb upstream from the Smp_331380 promoter site **(C)** Schematic of the 2kb upstream region of the *foxA* promoter with the sequences and position of the response element and primer set used. Length not proportional to the size. **(D)** ChIP-qPCR, shown as percentage of input DNA, for SmFTZ-F1 or preimmune serum antibodies at the suspected promoter for *foxA*. **(E)** ChIP-qPCR, shown as percentage of input DNA, for SmFTZ-F1 or preimmune serum antibodies at the putative promoter of *meg-8*.3 following RNAi treatment. Data are mean ± SEM from 3 biological replicates. *p<0.05 by Student’s t-test.
(TIFF)

S7 Fig *meg-8*.*3(RNAi) and Smftz-f1(RNAi)* animals do not accumulate dextran in their gut lumen. **(A)** FISH of *ctsb* (green) and fluorescent dextran (white) [46] in control(RNAi), *Smftz-f1(RNAi)* and *meg-8*.*3(RNAi)* animals at different time points. *n =* 3 biological replicates with >8 animals per replicate. Scale bars, 100 μm.
(TIFF)

S1 Table Oligonucleotide sequences used in this study. (XLSX)

S2 Table RNAseq analysis of transcriptional changes following *Smftz-f1(RNAi)* treatment. D5 and D9 tables represent entire datasets from Day 5 and Day 9 of RNAi, respectively. Subsequent tabs show subsets of data for the most down-regulated genes at D5 or D9 and the genes that were common between the D5 and D9 datasets.
(XLSX)

S3 Table qPCR values of 20 most differentially down regulated genes following *Smftz-f1(RNAi)* treatment. (XLSX)

S4 Table Response element sequences used in this study. Response elements that were tested as well as their relative binding affinity. Scores are–(no binding) to + (binding).
(XLSX)

S1 Video Movies showing control and *Smftz-f1(RNAi)* parasites at D20 of RNAi. (MOV)

S1 File Results of BLASTN search against v7 of the *S*. *mansoni* genome using sequences upstream of *meg-8*.*3* containing an FTZ-F1 response element as queries. (TXT)
