# Supplementary figures and images for: The Schistosoma mansoni nuclear receptor FTZ-F1 maintains esophageal gland function via transcriptional regulation of meg-8.3
Source: PLoS Pathog. 2021 Dec 15;17(12):e1010140. doi: 10.1371/journal.ppat.1010140 (PMC8673669; doi:10.1371/journal.ppat.1010140)

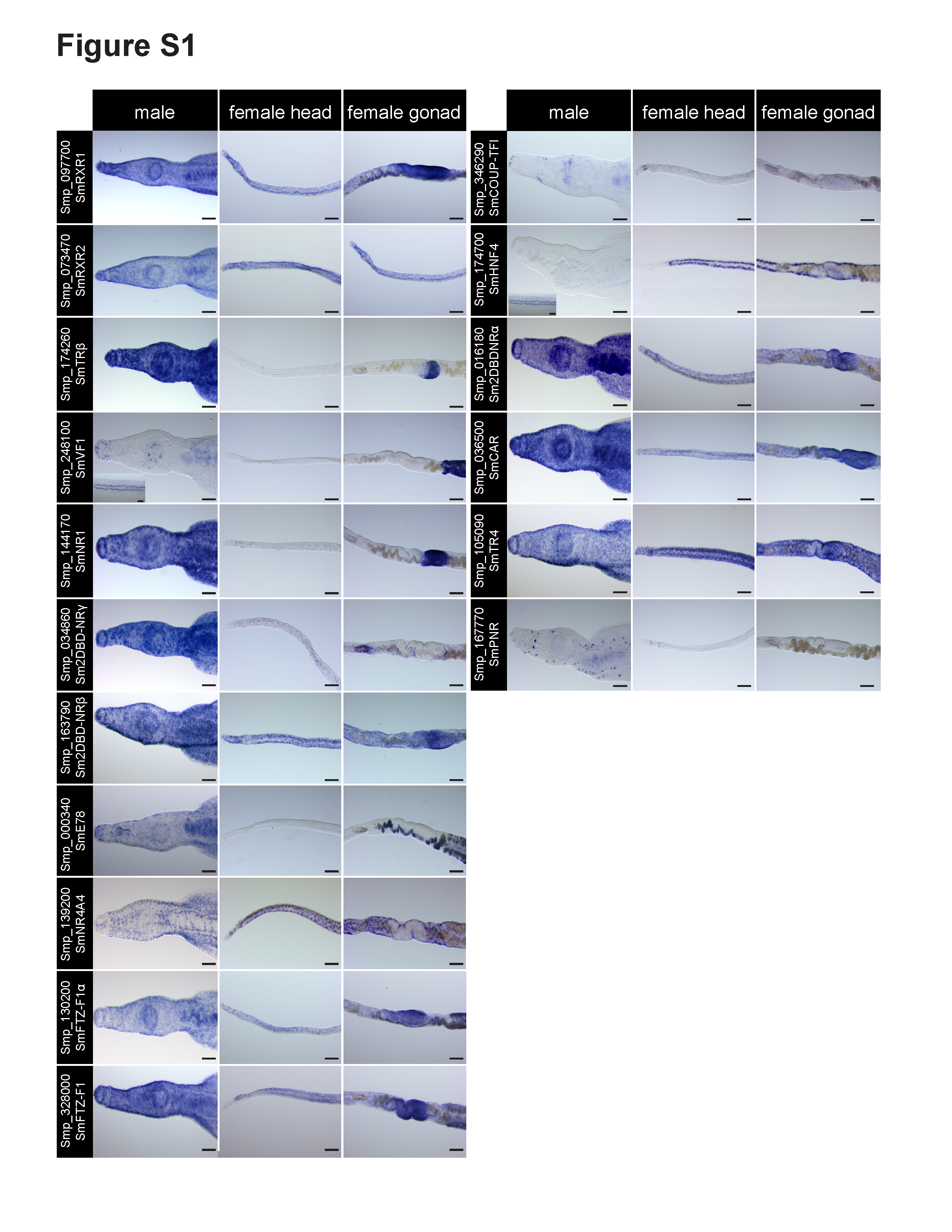

Supplement: S1 Fig — Whole mount in situ hybridization showing mRNA expression of NRs in male and female parasites. Anterior faces left. Representative of >10 animals from 3 biological replicates. Scale Bars: 100μm. (TIFF) [file ppat.1010140.s001.tiff]

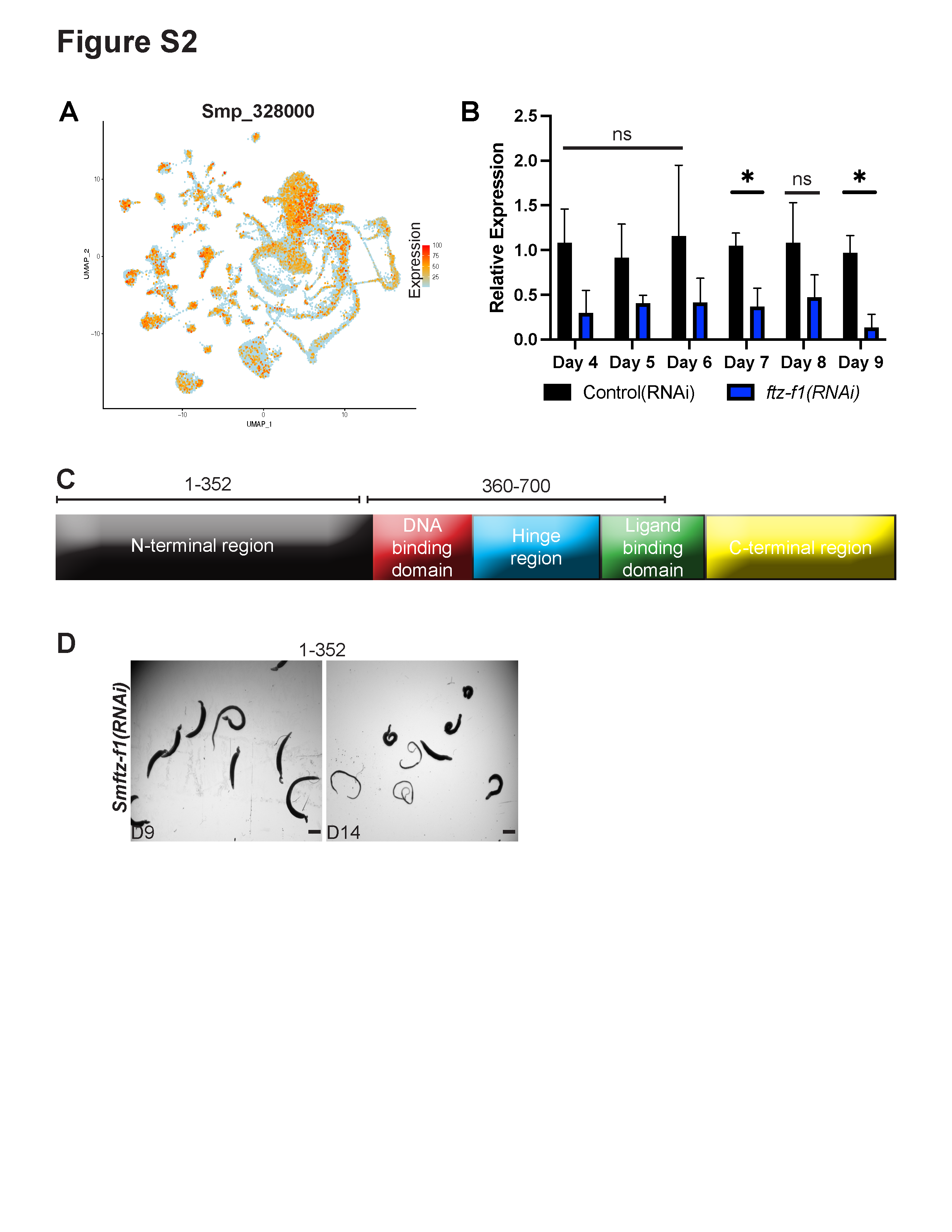

Supplement: S2 Fig — (A) UMAP plot of gene expression of SmFTZ-F1 (Smp_328000), plot generated using Schistocyte [23] (B) Graph of relative quantification of SmFTZ-F1 as determined by qPCR in either control(RNAi) or Smftz-f1(RNAi) animals. Data are mean ± SD from 3 biological replicates. *p<0.05 by Student’s t-test. (C) Cartoon of Smftz-f1 cDNA (top) and cDNA regions (in AA) targeted by two independent RNAi constructs (pAR22 and pAR31). pAR22 contains a cDNA fragment that spans from AA 360–700 of the smftz-f1 cDNA. pAR31 contains a cDNA fragment that spans from AA 1–352 of the smftz-f1 cDNA (D) Images of alternate Smftz-f1(RNAi) worms during in vitro culture. By D9 of in vitro culture, Smftz-f1(RNAi) animals fail to firmly attach to the bottom of the place. At D14, the worms are beginning to curl. Scale bar, 1 mm. (TIFF) [file ppat.1010140.s002.tiff]

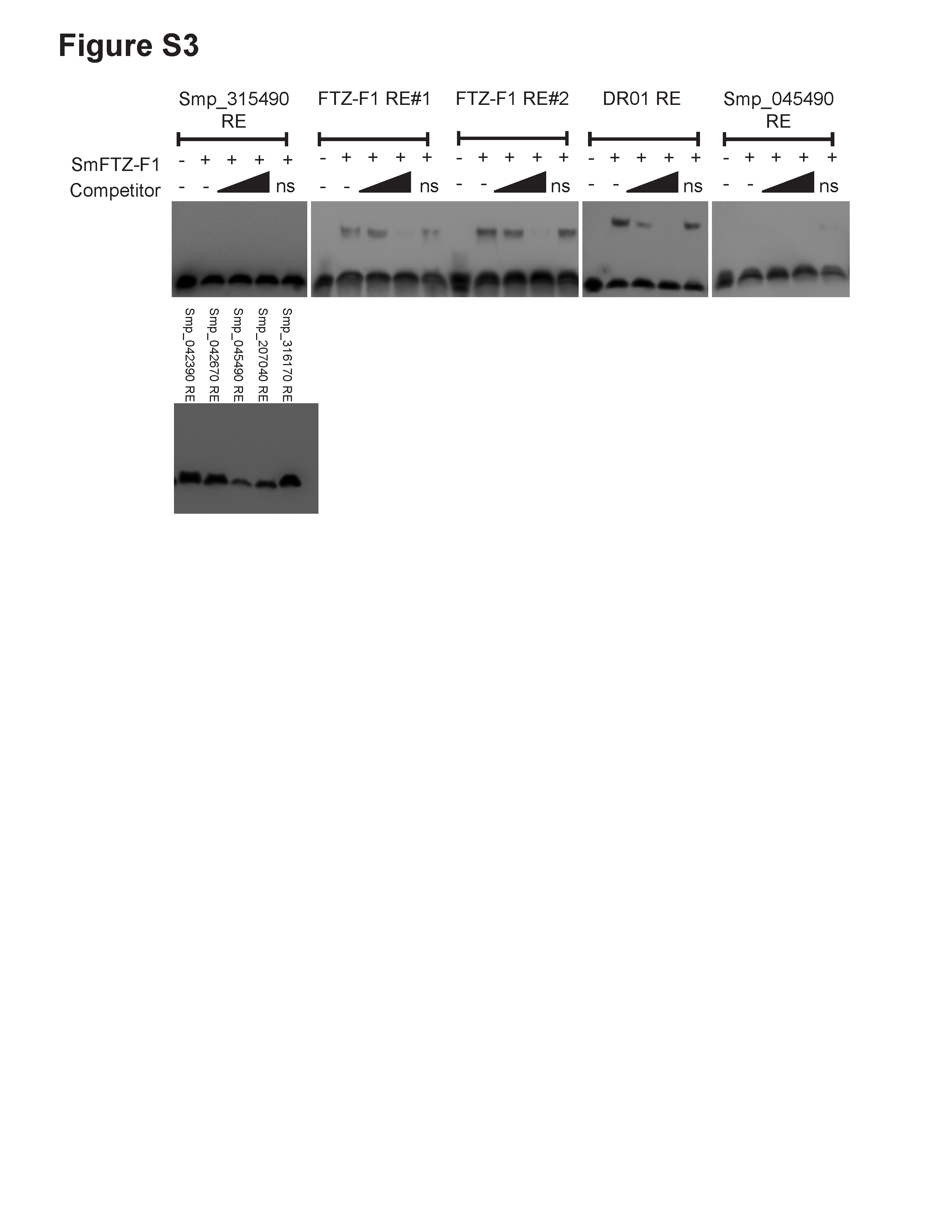

Supplement: S3 Fig — EMSAs were performed in presence or absence of in vitro translated SmFTZ-F1 protein ± the presence of 20-fold and 200-fold direct unlabeled competitor or 200-fold excess unlabeled competitor oligonucleotides. (TIFF) [file ppat.1010140.s003.tiff]

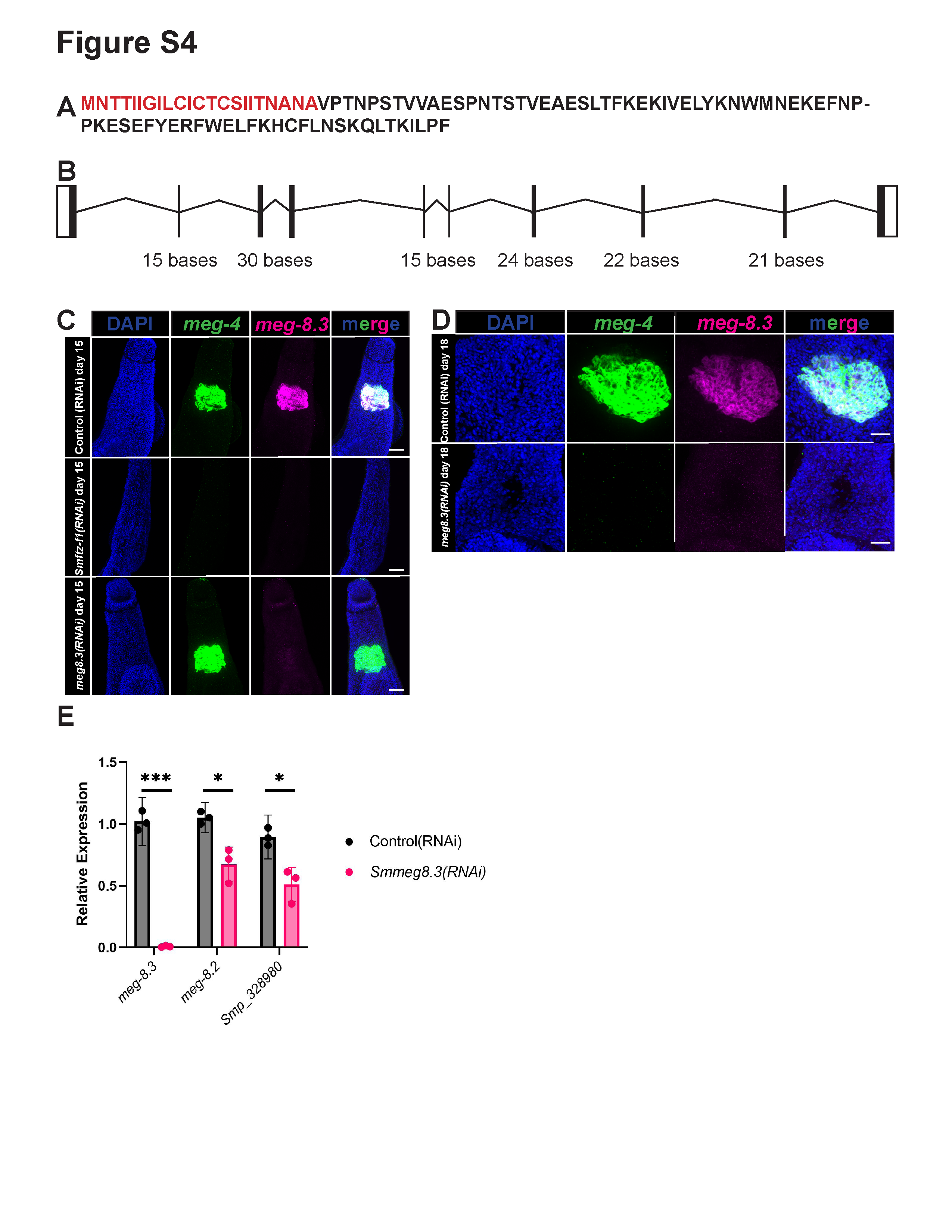

Supplement: S4 Fig — (A) Amino acid sequence of MEG-8.3. Red indicates a signal peptide according to SignalP (B) Schematic representation of gene structure of meg-8.3. Exon boxes are proportional to their lengths in bp. Lines represent introns shown with a length not proportional to their size. (C) Double FISH for meg-8.3 and meg-4 at day 15 for control(RNAi), Smftz-f1(RNAi), and meg-8.3(RNAi) parasites. Maximum intensity projections shown. n = 3 biological replicates of >5 worms per experiment. (D) Double FISH for meg-8.3 and meg-4 at day 18 for control(RNAi) and meg-8.3(RNAi) parasites. Maximum intensity projections shown. n = 1 replicate, 5 worms per treatment. (E) Graph of relative quantification of Smmeg-8.3, Smmeg-8.2, Smp_328980 (cystatin) as determined by qPCR in either control(RNAi) or Smmeg-8.3(RNAi) animals at Day 7 after the first dsRNA treatment. Data from 3 biological replicates. *p<0.05, **p<0.01, ***p<0.0001 by Student’s t-test. Error bars represent 95% confidence intervals. Scale bars: D, 50 μm. E, 25μm. (TIFF) [file ppat.1010140.s004.tiff]

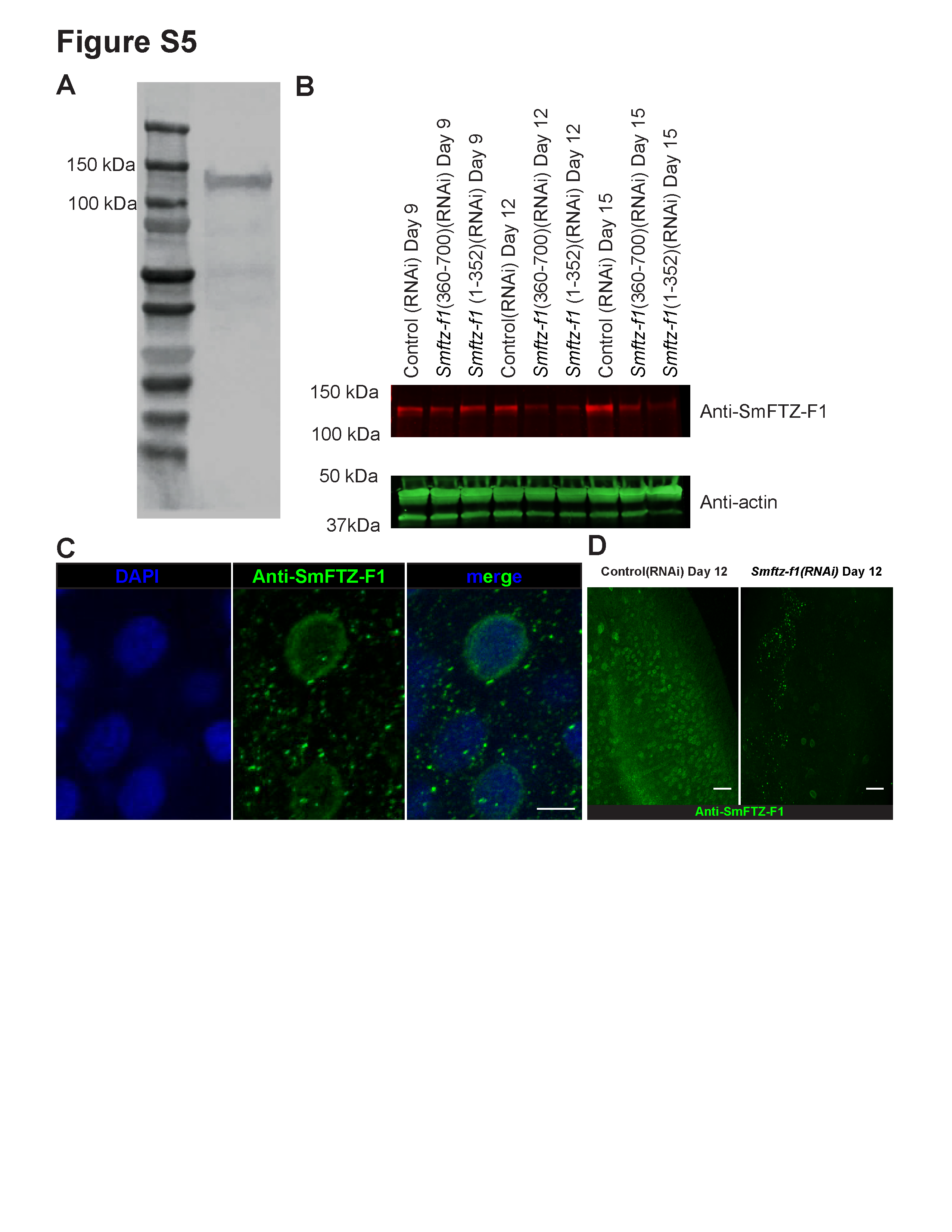

Supplement: S5 Fig — (A) Immunoblot of worm lysate testing the specificity of the SmFTZ-F1 antibody. Full length SmFTZ-F1 is predicted to be ~110 kD. (B) Western blot showing depletion of SmFTZ-F1 protein levels following Smftz-f1(RNAi). Actin labeling a positive control. n = 7 biological replicates taken from ~10 male worms/replicate (C) Immunofluorescence demonstrating that SmFTZ-F1 protein is found in the nuclei of cells. Maximum intensity projection (60X) is shown. n = 3 biological replicates of at least 5 worms per replicate. (D) Immunofluorescence of Day 12 control(RNAi) or Smftz-f1(RNAi) worms showing depletion of SmFTZ-F1+ cells n = 10 worms, 3 biological replicates. Scale bars: D, 5μm. E, 25μm. (TIFF) [file ppat.1010140.s005.tiff]

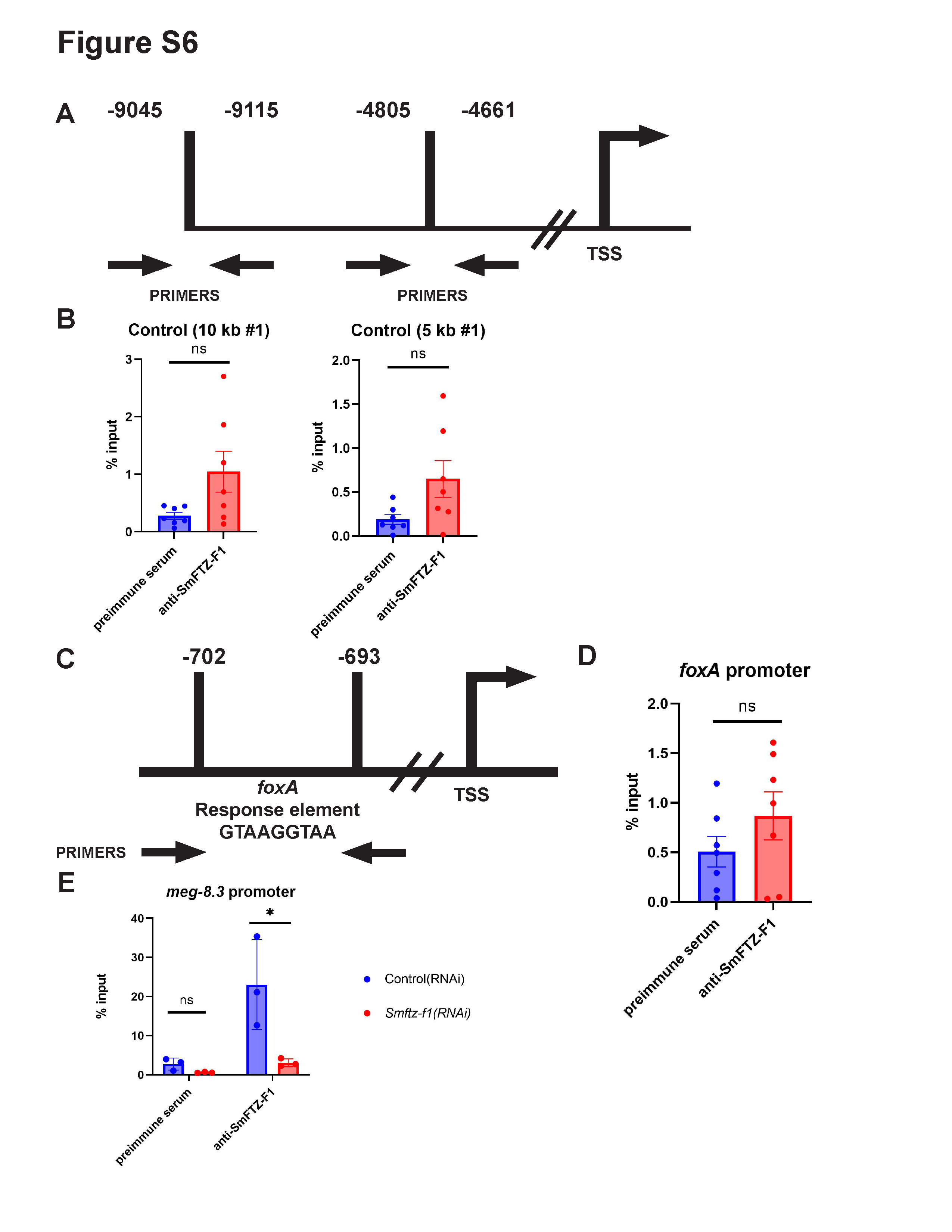

Supplement: S6 Fig — (A) Schematic of the 10kb upstream region of the Smp_331380 promoter with the position of the primer sets used. Length not proportional to the size. (B) ChIP-qPCR, shown as percentage of input DNA, for SmFTZ-F1 or preimmune serum antibodies at control regions 10kb or 5kb upstream from the Smp_331380 promoter site (C) Schematic of the 2kb upstream region of the foxA promoter with the sequences and position of the response element and primer set used. Length not proportional to the size. (D) ChIP-qPCR, shown as percentage of input DNA, for SmFTZ-F1 or preimmune serum antibodies at the suspected promoter for foxA. (E) ChIP-qPCR, shown as percentage of input DNA, for SmFTZ-F1 or preimmune serum antibodies at the putative promoter of meg-8.3 following RNAi treatment. Data are mean ± SEM from 3 biological replicates. *p<0.05 by Student’s t-test. (TIFF) [file ppat.1010140.s006.tiff]

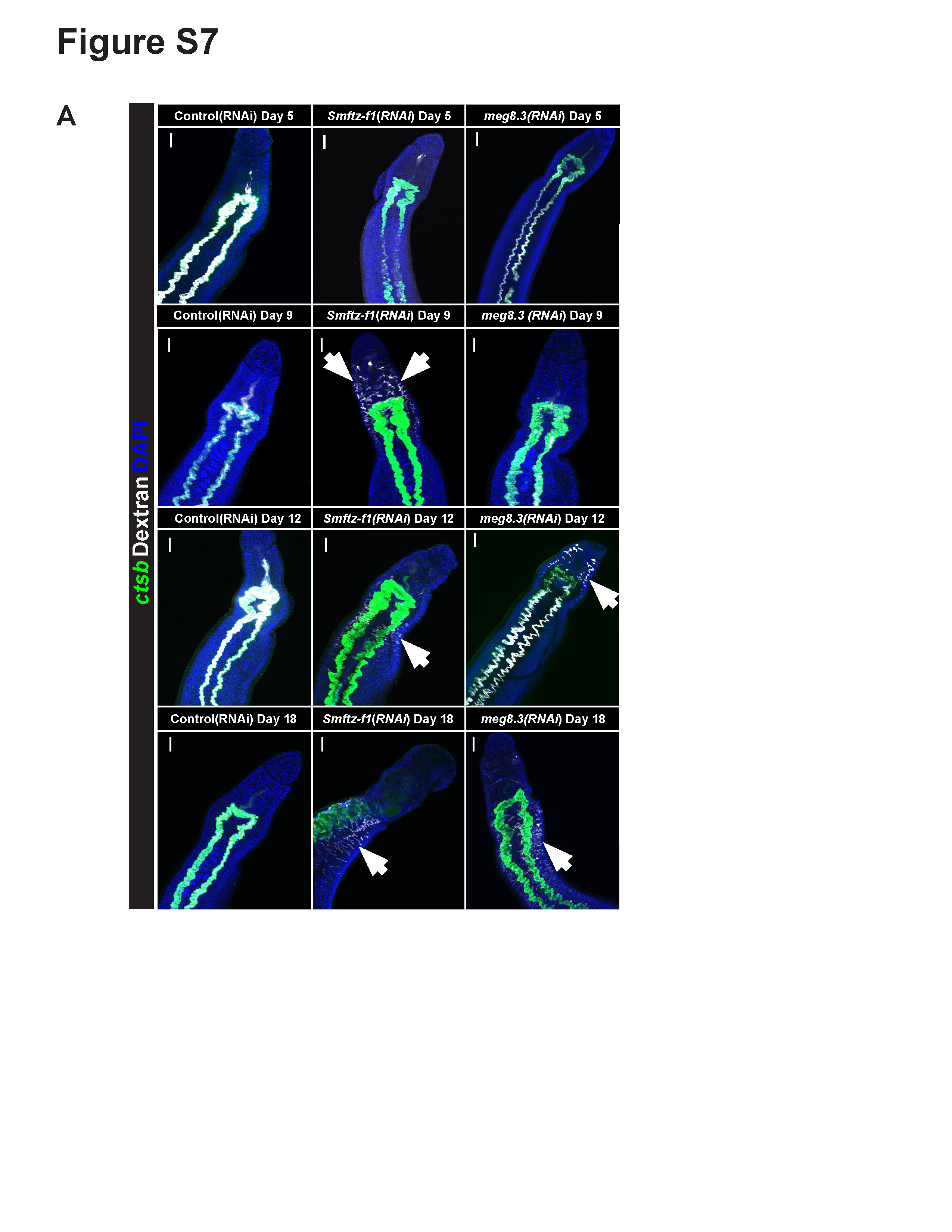

Supplement: S7 Fig — (A) FISH of ctsb (green) and fluorescent dextran (white) [46] in control(RNAi), Smftz-f1(RNAi) and meg-8.3(RNAi) animals at different time points. n = 3 biological replicates with >8 animals per replicate. Scale bars, 100 μm. (TIFF) [file ppat.1010140.s007.tiff]
